# Longer collapse-to-first-medical-contact interval is independently associated with ROSC in out-of-hospital cardiac arrest: a paradoxical finding that may reflect case-mix differences

**DOI:** 10.3389/fmed.2026.1989379

**Published:** 2026-10-27

**Authors:** Yixin Weng, Gaohaozhe Hu, Xuefeng Ma

**Affiliations:** Department of Emergency, Cixi Emergency Center, Ningbo, Zhejiang, China

**Keywords:** emergency medical services, first medical contact, out-of-hospital cardiac arrest, prehospital time interval, return of spontaneous circulation

## Abstract

**Background:**

Shortening prehospital time intervals is a cornerstone of out-of-hospital cardiac arrest (OHCA) management, with earlier medical contact generally assumed to improve survival. However, this assumption has rarely been scrutinized at the level of individual time metrics, particularly the interval from witnessed collapse to first medical contact (collapse-to-FMC), whose relationship with resuscitation outcomes independent of case-mix remains unclear. This study aimed to examine the association between core prehospital time intervals and the achievement of prehospital return of spontaneous circulation (ROSC).

**Patients and methods:**

This retrospective cohort study included 152 consecutive OHCA patients managed by the Cixi Emergency Center between April 2023 and March 2026. Time intervals were derived from synchronized dispatch center logs and onboard system timestamps, including dispatch-to-scene arrival, collapse-to-first medical contact, and collapse-to-CPR initiation intervals. Candidate variables for multivariable modeling were selected based on a prespecified causal rationale and clinical relevance, rather than solely on univariate statistical significance. Univariate logistic regression was performed for descriptive purposes to examine crude associations with prehospital ROSC. The main model included the two core time intervals (collapse-to-FMC and collapse-to-CPR initiation). Model 1 further adjusted for age, a clinically important confounder that did not reach statistical significance in univariate analysis. Model 2 additionally adjusted for etiology of arrest and presence of a shockable rhythm, which were selected *a priori* as pre-exposure confounders based on established prognostic importance in OHCA research. This approach ensured that clinically important confounders were retained regardless of their univariate *P* values. A main model included only time variables, and an adjusted model further incorporated clinical covariates. Multicollinearity was assessed using variance inflation factors, and model calibration was evaluated with the Hosmer–Lemeshow test. Discriminative ability was assessed by receiver operating characteristic curve analysis. Subgroup analyses were performed by age (<65 vs. ≥65 years) and etiology (cardiac vs. non-cardiac).

**Results:**

Among 152 patients, 52 (34.2%) achieved prehospital ROSC. Conventional dispatch-related intervals did not differ between groups. The collapse-to-first medical contact interval was significantly longer in the ROSC group (20.0 vs. 13.1 min, *P* < 0.001) and remained independently associated with ROSC after adjustment for prespecified pre-exposure confounders, including age, etiology of arrest, and presence of a shockable rhythm (OR 1.188, 95% CI: 1.079–1.307, *P* < 0.001). Bootstrap internal validation yielded an optimism-corrected AUC of 0.882 (95% CI: 0.827–0.936), with a calibration intercept of −0.063, a calibration slope of 0.972, and a Brier score of 0.132, indicating adequate calibration. In subgroup analyses, this association was predominantly observed in patients younger than 65 years (adjusted *P* = 0.037), and the interval from collapse to CPR initiation became significant in the cardiac etiology subgroup after adjustment (OR 1.567, 95% CI: 1.054–2.329, *P* = 0.026).

**Conclusion:**

In this cohort of OHCA patients, a longer collapse-to-first medical contact interval was paradoxically associated with increased odds of prehospital ROSC, independent of prespecified pre-exposure confounders. This counterintuitive finding suggests that the interval may reflect patient salvageability and unmeasured case-mix characteristics rather than treatment delay alone. These results challenge the conventional premise that shorter time to medical contact uniformly translates to better outcomes, and underscore the need for a more nuanced understanding of how patient characteristics shape the relationship between prehospital timing and resuscitation success.

## Introduction

Out-of-hospital cardiac arrest (OHCA) remains a leading cause of premature mortality worldwide, with reported incidence rates ranging from 50 to 90 per 100,000 person-years and overall survival to hospital discharge consistently below 10% in most Asian and European registries (1, 2). Since the Utstein consensus established standardized definitions in 1990, the dominant paradigm in resuscitation science has been that shorter prehospital time intervals linearly improve outcomes (3). This assumption is embedded in guidelines, benchmarking tools, and EMS quality indicators: dispatch activation within 1–2 min, ambulance response within 4–8 min, bystander CPR within 3–5 min, and defibrillation within 5–7 min are treated as non-negotiable targets (4). Large registry analyses from Japan, Korea, and Sweden have indeed shown that each 1-min increase in EMS response time reduces the adjusted odds of neurologically intact survival by 10%–15%, and that collapse-to-call delay is independently associated with lower survival (5–8). In this framework, the interval from witnessed collapse to first medical contact (collapse-to-FMC) is implicitly assumed to behave identically: the sooner a physician touches the patient, the better the chance of return of spontaneous circulation (ROSC).

However, this linear “time is muscle” narrative rests almost entirely on studies that use survival to hospital discharge or 30-day neurologic outcome as endpoints, while collapsing all prehospital intervals into a single response-time metric (9). Few investigations have isolated the collapse-to-FMC interval as an independent exposure, and fewer still have examined its relation to the proximal endpoint of prehospital ROSC, which is physiologically closer to the resuscitative act itself and less confounded by in-hospital decisions (10). The distinction matters because ROSC and survival to discharge are not interchangeable: a patient who achieves prehospital ROSC may still die from post-arrest brain injury, while a patient with sustained ROSC after prolonged on-scene effort may carry a different case-mix than one who is declared dead at scene (11, 12). Two mechanistic possibilities therefore remain underexplored. First, FMC time may not be a pure marker of system delay but a composite that includes patient-side delay (collapse recognition, call activation), bystander behavior, and the resuscitator's decision to invest time in a potentially salvageable patient. Second, the direction of association may be reversed in specific subgroups if longer FMC selectively captures arrests with reversible etiology (e.g., asphyxial, toxic, or young patients with prolonged salvage window) in whom on-scene effort is more intense and more often successful (13, 14). Whether the collapse-to-FMC interval is an independent predictor of prehospital ROSC, and whether its direction survives adjustment for prespecified pre-exposure confounders, has not been examined in a single-system Chinese EMS cohort with synchronized dispatch and onboard timestamps.

In this study, we analyzed 152 consecutive OHCA patients managed by the Cixi Emergency Center between April 2023 and March 2026. Time intervals were derived from synchronized dispatch center logs and ambulance onboard timestamps, allowing the collapse-to-first medical contact (collapse-to-FMC) interval to be examined as a distinct exposure rather than a component of aggregate response time. We assessed its association with prehospital ROSC after multivariable adjustment for prespecified pre-exposure confounders, including age, arrest etiology, and presence of a shockable rhythm, and further explored whether this association varied across age and etiology subgroups. By anchoring the outcome to prehospital ROSC and isolating a single time metric, this study sought to determine whether prehospital time intervals should be interpreted as direct causal levers or as markers shaped by case-mix and on-scene clinical decisions in a single-system Chinese EMS cohort.

## Materials and methods

### Study design and population

This retrospective observational study was conducted at Cixi Emergency Center, a prehospital emergency medical service (EMS) agency serving a population of approximately 1.8 million residents in Cixi, Zhejiang Province, China. Consecutive patients who experienced out-of-hospital cardiac arrest (OHCA) and were managed by our emergency physicians between April 2023 and March 2026 were screened for eligibility. Patients were included if they met the following criteria: (1) OHCA confirmed by absence of spontaneous breathing and palpable pulse upon emergency physician arrival; (2) resuscitation attempted by the EMS team, either manual cardiopulmonary resuscitation (CPR) or defibrillation; (3) complete documentation of the emergency call timeline, including dispatch time, scene arrival time, and first medical contact time. Patients were excluded if (1) the arrest was witnessed by EMS personnel (i.e., arrest occurred after physician contact); (2) resuscitation was withheld due to obvious signs of death, do-not-resuscitate orders, or terminal illness; (3) the prehospital medical records were incomplete for key time variables. Of the 211 initially identified records, 177 were assessed for eligibility after excluding 34 non-eligible records (e.g., unconfirmed OHCA and duplicate logs). Following the exclusion of 25 patients during eligibility assessment, a total of 152 patients were ultimately included in the analysis (Figure 1). The study was approved by the institutional review board of Cixi Emergency Center, and the requirement for informed consent was waived due to the retrospective, de-identified nature of the data.

**Figure 1:**
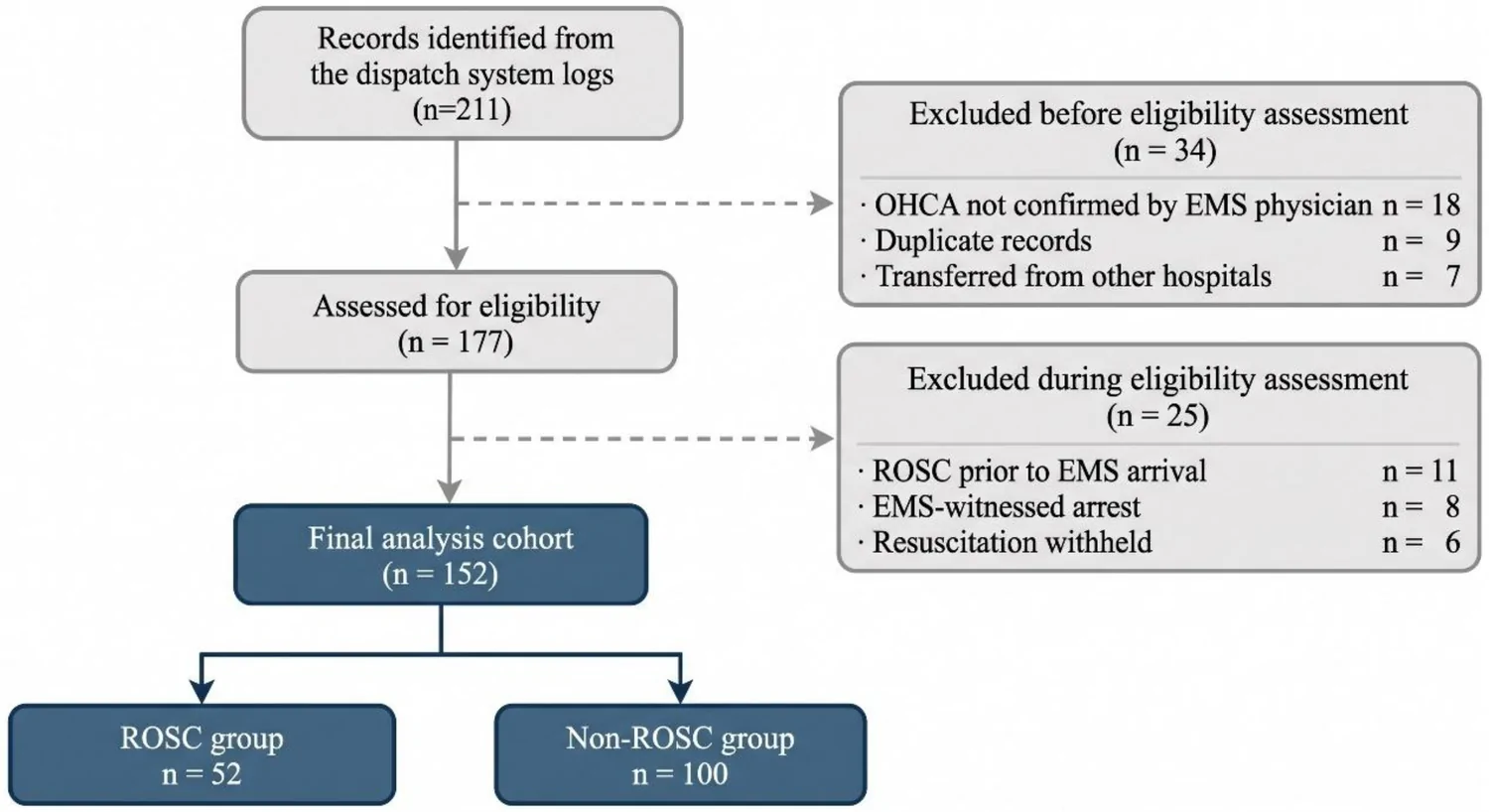
Flowchart of patient selection and study cohort composition. Of 211 records identified from dispatch logs, 34 were excluded before eligibility assessment (unconfirmed OHCA, *n* = 22; duplicate logs, *n* = 12), and 25 were excluded during eligibility assessment (EMS-witnessed arrest, *n* = 8; resuscitation withheld, *n* = 9; incomplete records, *n* = 8). The final analysis included 152 patients, divided into the ROSC group (*n* = 52) and non-ROSC group (*n* = 100). OHCA, out-of-hospital cardiac arrest; EMS, emergency medical services; ROSC, return of spontaneous circulation.

For each patient, prehospital medical records and dispatch system logs were reviewed to extract demographic characteristics, preexisting comorbidities, arrest etiology, witness status, bystander CPR and AED use, dispatch-to-arrival intervals, first medical contact time, CPR initiation time, airway management, vascular access, defibrillation episodes, and adrenaline administration. The primary outcome was prehospital return of spontaneous circulation (ROSC), defined as the restoration of a palpable pulse and measurable blood pressure sustained for at least 20 s without ongoing chest compressions, achieved at any point during prehospital resuscitation, and confirmed by the attending emergency physician. This 20-s threshold follows convention in cardiac arrest reporting, where ROSC denotes any recovery of a spontaneous perfusing rhythm regardless of duration, while sustained ROSC is conventionally defined as circulation without chest compressions for 20 consecutive minutes (15). Our definition therefore captures any prehospital ROSC, including brief or transient ROSC, and was chosen because prehospital records in this EMS cohort documented pulse/blood pressure episodes but not continuous post-ROSC hemodynamics. It should not be interpreted as sustained ROSC, survival to hospital arrival, survival to discharge, or favorable neurologic outcome. Based on this outcome, patients were divided into two groups: the ROSC group (*n* = 52) and the non-ROSC group (*n* = 100). All time intervals were derived from synchronized dispatch center logs and ambulance onboard system timestamps, and were calculated as follows: (1) the call-to-dispatch interval was calculated from the timestamp of the incoming emergency call to the timestamp of vehicle dispatch; (2) the dispatch-to-scene interval was calculated from the timestamp of vehicle dispatch to the moment the ambulance stopped at the scene location; (3) the EMS arrival time was calculated from the timestamp of the incoming emergency call to the moment the ambulance stopped at the scene; (4) the scene-to-FMC interval was calculated from the moment the ambulance stopped at the scene to the moment the emergency physician first physically touched the patient; (5) the collapse-to-FMC interval, the primary exposure of this study, was calculated from the witnessed collapse time (or estimated collapse time for unwitnessed arrests) to the moment the emergency physician first physically touched the patient, including palpation of the carotid pulse, connection of the monitor, or initiation of chest compressions, whichever occurred first; and (6) the collapse-to-CPR initiation interval was calculated from the witnessed or estimated collapse time to the moment the first chest compression was delivered by any rescuer, whether a bystander or an EMS provider. The collapse time was obtained from witness or bystander reports at the scene, or estimated by the attending emergency physician when the arrest was unwitnessed. All intervals were expressed in minutes.

### Data collection and definitions

Data were collected retrospectively from two integrated sources within the Cixi emergency medical dispatch system. The first source was the dispatch center log, which automatically recorded the timestamp of each incoming call, vehicle dispatch, scene arrival, and hospital handover. The second source was the standardized prehospital medical record completed by the attending emergency physician at the scene, which documented patient demographics, arrest characteristics, interventions performed, and time points not captured by the dispatch system, including first medical contact and CPR initiation. All time records were generated automatically by the dispatch system or manually entered by the physician at the moment each event occurred, and were cross-validated against audio recordings of the dispatch communications when discrepancies exceeding 1 min were identified. Data extraction was performed by two trained emergency physicians independently, and disagreements were resolved by consensus with a third senior physician.

The collapse-to-FMC interval was calculated as the time from the witnessed collapse (or estimated collapse time for unwitnessed arrests) to the moment the emergency physician first physically touched the patient. The collapse time was obtained from witness or bystander reports, or estimated by the attending physician when unwitnessed. Scene arrival time was defined as the moment the ambulance stopped at the scene location. First medical contact time was defined as the moment the emergency physician first physically touched the patient, including palpation of the carotid pulse, connection of the monitor, or initiation of chest compressions, whichever occurred first. CPR initiation time was defined as the moment the first chest compression was delivered by any rescuer, whether a bystander or an EMS provider. Dispatch-to-scene arrival time was calculated from the emergency call timestamp to scene arrival. The collapse-to-FMC and collapse-to-CPR initiation intervals were calculated from the witnessed or estimated collapse time to the respective event. All intervals were expressed in minutes. For unwitnessed arrests, the collapse time was estimated by the attending emergency physician based on the available history and scene information.

Demographic and pre-arrest characteristics included age, sex, preexisting comorbidities, and the etiology of the arrest, which was classified as cardiac or non-cardiac based on the physician's clinical judgment at the scene. Witnessed arrest was defined as the collapse being directly observed or heard by another person. Bystander CPR was defined as chest compressions initiated by a non-EMS rescuer prior to the arrival of the EMS team. Shockable rhythm was defined as ventricular fibrillation or pulseless ventricular tachycardia documented on the first electrocardiographic rhythm analysis. Defibrillation was defined as the delivery of a synchronized or non-synchronized electrical shock. Airway management included endotracheal intubation, supraglottic airway insertion, and bag-valve-mask ventilation. Vascular access was recorded if the physician attempted or successfully established intravenous access. Adrenaline administration was recorded with respect to dosage and number of doses. The primary outcome was prehospital return of spontaneous circulation (ROSC), defined as the restoration of a palpable pulse and measurable blood pressure sustained for at least 20 s without ongoing chest compressions, achieved at any point during prehospital resuscitation and confirmed by the attending emergency physician. Patients were categorized into the ROSC group or the non-ROSC group based on this outcome.

### Statistical analysis

Continuous variables were tested for normality using the Shapiro–Wilk test. Non-normally distributed data were expressed as median with interquartile range and compared between groups using the Mann–Whitney *U* test. Categorical variables were expressed as counts with percentages and compared using the chi-square test or Fisher's exact test, as appropriate. Prior to analysis, missing data were assessed for pattern and proportion. Among the analyzed variables, age had 20 missing values (13.2%) and sex had 5 missing values (3.3%); all other variables were complete. No variable exceeded the 20% missingness threshold, and no variables were excluded. Missing values were handled using multiple imputation by chained equations with 5 imputed datasets and 10 iterations per dataset, under the assumption of missing at random. The imputation model included all analysis variables (age, sex, etiology of arrest, shockable rhythm, collapse-to-FMC interval, collapse-to-CPR initiation interval, and the outcome variable ROSC) as predictors. Convergence was assessed by inspecting trace plots of the imputed values across iterations, which showed no systematic trends. Estimates from the 5 imputed datasets were pooled using Rubin's rules. Sensitivity analyses comparing complete-case and imputed datasets yielded consistent results, supporting the robustness of the imputation approach.

To avoid sparse-data bias and model instability, a clinically prespecified parsimonious modeling strategy was adopted rather than univariate significance-based variable selection. The main model included only the two core time intervals (collapse-to-FMC and collapse-to-CPR initiation intervals), yielding 2 parameters with an events-per-variable (EPV) ratio of 26. Model 1 additionally adjusted for age (3 parameters, EPV = 17.3). Model 2 further adjusted for age, etiology of arrest, and presence of a shockable rhythm (5 parameters, EPV = 10.4). Post-exposure interventions (airway management, intravenous access, adrenaline administration, and clinical pathway activation) were not included in the primary analysis, as these variables occur after first medical contact and were therefore considered mediators on the causal pathway from collapse-to-FMC to ROSC rather than confounders. Adjusting for these post-exposure variables would introduce overadjustment bias by blocking the indirect effect of the exposure on the outcome. In contrast, age, etiology of arrest, and presence of a shockable rhythm were specified *a priori* as pre-exposure confounders, as they precede the collapse-to-FMC interval and may independently influence both the timing of medical contact and the likelihood of ROSC. The two core time intervals (collapse-to-FMC and collapse-to-CPR initiation) were both included in the main model because they capture distinct components of the prehospital timeline: the former reflects the interval from collapse to professional contact, while the latter reflects the total no-flow period prior to resuscitation. Although these intervals are correlated (VIF = 3.7), multicollinearity diagnostics confirmed that simultaneous inclusion was acceptable. All EPV values exceeded the recommended threshold of 10. To further evaluate the stability of the estimated odds ratios and confidence intervals, bootstrap internal validation with 1,000 resamples was performed, which yielded an optimism-corrected AUC of 0.882 (95% CI: 0.827–0.936) and a calibration slope of 0.972, indicating minimal overfitting and stable model performance. A main model was constructed including only the two core time intervals (collapse-to-FMC and collapse-to-CPR initiation). Adjusted models were further built with the addition of prespecified pre-exposure confounders, including age (Model 1), and age, etiology of arrest, and presence of a shockable rhythm (Model 2). Multicollinearity was assessed using tolerance and the variance inflation factor. Model fit was evaluated using the Hosmer–Lemeshow goodness-of-fit test. To further assess calibration, bootstrap internal validation with 1,000 resamples was performed to obtain optimism-corrected discrimination, calibration intercept and slope, a calibration plot, and the Brier score. The discriminatory ability of the final model was assessed using the area under the receiver operating characteristic curve, with sensitivity, specificity, optimal cutoff value, and Youden index determined. Probability thresholds were defined based on the Youden index. Subgroup analyses were performed by age (<65 vs. ≥65 years) and etiology of arrest (cardiac vs. non-cardiac). To assess potential nonlinearity of the time variables, sensitivity analyses were conducted by including quadratic terms for the collapse-to-FMC and collapse-to-CPR initiation intervals in the main model; no statistically significant nonlinear effects were detected, and the linear specification was retained for parsimony and interpretability. All analyses were performed using SPSS version 27.0 (IBM Corp., Armonk, NY, USA). A two-tailed *P* < 0.05 was considered statistically significant.

## Results

### Baseline characteristics of the study population

A total of 152 patients with out-of-hospital cardiac arrest were included, of whom 52 (34.2%) achieved prehospital ROSC and 100 (65.8%) did not. The demographic and clinical characteristics of the two groups are summarized in Table 1. The median age was 61 years in both groups (*P* = 0.953), and the proportion of male patients was slightly higher in the non-ROSC group (69.0% vs. 61.5%), although the difference was not statistically significant (*P* = 0.355). Among preexisting comorbidities, diabetes mellitus was significantly more prevalent in the ROSC group (7.7% vs. 1.0%, *P* = 0.047); no significant differences were observed for stroke, hypertension, hyperlipidemia, or coronary artery disease (all *P* > 0.05). The etiology of arrest differed markedly between groups: non-cardiac causes accounted for 71.2% of the ROSC group but only 22.0% of the non-ROSC group (*P* < 0.001). A shockable initial rhythm, indicated by the presence of a defibrillation indication, was more frequent in the ROSC group (30.8% vs. 16.0%, *P* = 0.034). No significant differences were found in the proportion of witnessed arrests, bystander CPR, or telephone-assisted medical instructions between the two groups (all *P* > 0.05).

**Table 1:** Baseline characteristics of patients with out-of-hospital cardiac arrest stratified by resuscitation outcome.

Characteristic	Non-ROSC (*n* = 100)	ROSC (*n* = 52)	*χ*^2^/*Z*	*P*
Age, years	61 (51, 74)	61 (52.5, 73)	−0.058	0.953
Male, *n* (%)	69 (69.0)	32 (61.5)	0.854	0.355
Comorbidities, *n* (%)				
Stroke	10 (10.0)	3 (5.8)	0.783	0.376
Hypertension	9 (9.0)	4 (7.7)	0.000	1.000
Hyperlipidemia	9 (9.0)	4 (7.7)	0.000	1.000
Diabetes mellitus	1 (1.0)	4 (7.7)	–	0.047
Coronary artery disease	8 (8.0)	3 (5.8)	0.030	0.862
Etiology of arrest, *n* (%)			34.804	<0.001
Cardiac	78 (78.0)	15 (28.8)		
Non-cardiac	22 (22.0)	37 (71.2)		
Witnessed arrest, *n* (%)	45 (45.0)	23 (44.2)	0.008	0.928
Bystander CPR, *n* (%)	45 (45.0)	23 (44.2)	0.008	0.928
Telephone-assisted instruction, *n* (%)	28 (28.0)	17 (32.7)	0.361	0.548
Shockable initial rhythm, *n* (%)	16 (16.0)	16 (30.8)	4.490	0.034

Continuous data are presented as median (interquartile range). Categorical data are presented as *n* (%). *P* values were derived from the Mann–Whitney *U* test for continuous variables and the chi-square test or Fisher's exact test (when expected cell count <5) for categorical variables. ROSC, return of spontaneous circulation; CPR, cardiopulmonary resuscitation.

### Comparison of prehospital interventions between groups

Table 2 summarizes the prehospital interventions performed in the two groups. The proportion of patients who received defibrillation was higher in the ROSC group (28.8% vs. 16.0%), although the difference did not reach statistical significance (*P* = 0.062). Endotracheal intubation was performed more frequently in the ROSC group (34.6% vs. 9.0%, *P* < 0.001). Intravenous access was attempted in 90.4% of patients in the ROSC group compared with 37.0% in the non-ROSC group (*P* < 0.001), and successful access was achieved in 84.6% vs. 32.0%, respectively (*P* < 0.001). Adrenaline was administered to 76.9% of ROSC patients and 30.0% of non-ROSC patients (*P* < 0.001). Adequate on-scene resuscitation was documented in 44.2% of the ROSC group vs. 13.0% of the non-ROSC group (*P* < 0.001). Notably, 98.1% of ROSC patients were managed according to the clinical pathway, compared with 70.0% of non-ROSC patients (*P* < 0.001).

**Table 2:** Prehospital interventions in patients with out-of-hospital cardiac arrest stratified by resuscitation outcome.

Intervention	Non-ROSC (*n* = 100)	ROSC (*n* = 52)	*χ* ^2^	*P*
Defibrillation, *n* (%)	16 (16.0)	15 (28.8)	3.477	0.062
Endotracheal intubation, *n* (%)	9 (9.0)	18 (34.6)	15.366	<0.001
Intravenous access attempted, *n* (%)	37 (37.0)	47 (90.4)	39.436	<0.001
Intravenous access successful, *n* (%)	32 (32.0)	44 (84.6)	37.883	<0.001
Adrenaline administration, *n* (%)	30 (30.0)	40 (76.9)	30.319	<0.001
Adequate on-scene resuscitation, *n* (%)	13 (13.0)	23 (44.2)	18.461	<0.001
Clinical pathway activation, *n* (%)	70 (70.0)	51 (98.1)	16.611	<0.001

Data are presented as *n* (%). *P* values were derived from the chi-square test. ROSC, return of spontaneous circulation.

### Comparison of time intervals between groups

Table 3 presents the time-related metrics of the two groups. No significant differences were observed between the ROSC and non-ROSC groups regarding EMS arrival time (13.0 vs. 11.9 min, *P* = 0.347), call-to-dispatch time (2.0 vs. 1.7 min, *P* = 0.712), dispatch-to-scene time (10.0 vs. 9.8 min, *P* = 0.389), or scene-to-first-medical-contact time (1.0 vs. 0.03 min, *P* = 0.138). However, the collapse-to-first medical contact interval was significantly longer in the ROSC group than in the non-ROSC group (20.0 vs. 13.1 min, *P* < 0.001). Similarly, the collapse-to-CPR initiation interval was significantly longer in the ROSC group (20.5 vs. 14.6 min, *P* < 0.001). These findings indicate that patients who achieved ROSC experienced longer prehospital time intervals prior to the initiation of resuscitation, a seemingly paradoxical relationship that warrants further examination in the multivariable analysis.

**Table 3:** Prehospital time intervals in patients with out-of-hospital cardiac arrest stratified by resuscitation outcome.

Time interval	Non-ROSC (*n* = 100)	ROSC (*n* = 52)	*Z*	*P*
EMS arrival time, min	11.93 (8.51, 18.22)	13.00 (10.50, 17.50)	−0.940	0.347
Collapse-to-FMC interval, min	13.10 (10.22, 18.55)	20.00 (15.50, 23.50)	−4.785	<0.001
Collapse-to-CPR initiation interval, min	14.64 (10.97, 20.65)	20.50 (17.00, 27.00)	−4.777	<0.001
Call to dispatch, min	1.73 (1.23, 2.36)	2.00 (1.00, 3.00)	−0.369	0.712
Dispatch to scene, min	9.83 (6.92, 14.98)	10.00 (8.00, 13.00)	−0.862	0.389
Scene-to-FMC interval, min	0.03 (0, 1.23)	1.00 (0, 2.00)	−1.485	0.138

Data are presented as median (interquartile range). *P* values were derived from the Mann–Whitney *U* test. ROSC, return of spontaneous circulation; EMS, emergency medical services; CPR, cardiopulmonary resuscitation.

### Univariate logistic regression analysis

Univariate binary logistic regression was performed to describe the crude association of each candidate variable with prehospital ROSC. Variables were selected for multivariable modeling based on a prespecified causal rationale and clinical relevance, as described in the Statistical Analysis section. As shown in Table 4, several variables demonstrated significant associations with the outcome. Patients with non-cardiac etiology of arrest had significantly higher odds of ROSC compared with those with cardiac etiology (OR 8.745, 95% CI: 4.073–18.776, *P* < 0.001). The presence of a defibrillation indication was associated with increased odds of ROSC (OR 2.333, 95% CI: 1.053–5.169, *P* = 0.037). Endotracheal intubation (OR 5.353, 95% CI: 2.194–13.06, *P* < 0.001), intravenous access attempt (OR 16.005, 95% CI: 5.845–43.83, *P* < 0.001), successful intravenous access (OR 11.687, 95% CI: 4.933–27.69, *P* < 0.001), and adrenaline administration (OR 7.778, 95% CI: 3.587–16.866, *P* < 0.001) were all significantly associated with higher odds of achieving ROSC. Adequate on-scene resuscitation (OR 5.308, 95% CI: 2.386–11.806, *P* < 0.001) and clinical pathway activation (OR 21.857, 95% CI: 2.886–165.555, *P* = 0.003) also showed significant associations. Among time-related variables, only the collapse-to-first medical contact interval was significantly associated with ROSC (OR 1.080, 95% CI: 1.028–1.135, *P* = 0.002). No significant associations were observed for age, sex, comorbidities (diabetes mellitus, *P* = 0.062), witnessed arrest, bystander CPR, telephone-assisted instruction, defibrillation, or the remaining time intervals (all *P* > 0.05).

**Table 4:** Univariate binary logistic regression analysis of factors associated with prehospital ROSC.

Variable	*B*	SE	Wald	*P*	OR	95% CI
Sex (male vs. female)	−0.330	0.358	0.851	0.356	0.719	0.357–1.449
Age, years	−0.002	0.010	0.028	0.867	0.998	0.978–1.019
Stroke	−0.596	0.682	0.764	0.382	0.551	0.145–2.097
Hypertension	−0.171	0.627	0.075	0.785	0.843	0.247–2.879
Hyperlipidemia	−0.171	0.627	0.075	0.785	0.843	0.247–2.879
Diabetes mellitus	2.110	1.132	3.476	0.062	8.250	0.898–75.828
Coronary artery disease	−0.351	0.700	0.251	0.616	0.704	0.179–2.775
Etiology of arrest (non-cardiac vs. cardiac)	2.169	0.390	30.944	<0.001	8.745	4.073–18.776
Witnessed arrest	−0.031	0.344	0.008	0.928	0.969	0.494–1.903
Bystander CPR	−0.031	0.344	0.008	0.928	0.969	0.494–1.903
Telephone-assisted instruction	0.222	0.370	0.361	0.548	1.249	0.605–2.580
Defibrillation indication	0.847	0.406	4.359	0.037	2.333	1.053–5.169
Defibrillation performed	0.755	0.410	3.394	0.065	2.128	0.953–4.754
Endotracheal intubation	1.678	0.455	13.592	<0.001	5.353	2.194–13.060
Intravenous access attempted	2.773	0.514	29.106	<0.001	16.005	5.845–43.830
Intravenous access successful	2.459	0.440	31.207	<0.001	11.687	4.933–27.690
Adrenaline administration	2.051	0.395	26.981	<0.001	7.778	3.587–16.866
Adequate on-scene resuscitation	1.669	0.408	16.745	<0.001	5.308	2.386–11.806
Clinical pathway activation	3.085	1.033	8.915	0.003	21.857	2.886–165.555
EMS arrival time, min	0.011	0.023	0.221	0.639	1.011	0.966–1.058
Collapse-to-FMC interval, min	0.077	0.025	9.407	0.002	1.080	1.028–1.135
Collapse-to-CPR initiation interval, min	0.024	0.014	2.943	0.086	1.024	0.997–1.052
Call to dispatch, min	−0.020	0.055	0.137	0.711	0.980	0.881–1.091
Dispatch to scene, min	0.020	0.027	0.562	0.453	1.020	0.968–1.075
Scene-to-FMC interval, min	0.036	0.045	0.647	0.421	1.036	0.950–1.131

Data are presented as odds ratio (OR) with 95% confidence interval (CI). Univariate binary logistic regression was performed with prehospital ROSC as the dependent variable. ROSC, return of spontaneous circulation; EMS, emergency medical services; CPR, cardiopulmonary resuscitation.

### Multivariable logistic regression and model performance

#### Multivariable logistic regression analysis

Three multivariable logistic regression models were constructed to examine the independent association between core time intervals and prehospital ROSC. The main model included only the two core time intervals. Model 1 additionally adjusted for age. Model 2 further adjusted for etiology of arrest and presence of a shockable rhythm. Post-exposure interventions were not included in the primary analysis, as these variables may act as mediators rather than confounders. Collinearity diagnostics showed that all tolerance values exceeded 0.1 and all variance inflation factors (VIF) were below 10, indicating no severe multicollinearity among the included variables (Table 5).

**Table 5:** Multivariable logistic regression analysis of core time intervals with prespecified pre-exposure confounders.

Variable	*B*	SE	Wald	*P*	OR	95% CI	Tolerance	VIF
Main model								
Collapse-to-FMC interval, min	0.128	0.040	10.328	0.001	1.136	1.051–1.228	0.269	3.718
Collapse-to-CPR initiation interval, min	−0.033	0.019	2.977	0.084	0.967	0.931–1.005	0.269	3.718
Main model + Covariate 1								
Collapse-to-FMC interval, min	0.128	0.040	10.295	0.001	1.136	1.051–1.229	0.269	3.720
Collapse-to-CPR initiation interval, min	−0.033	0.019	2.975	0.085	0.967	0.931–1.005	0.269	3.721
Age, years	−0.011	0.377	0.001	0.976	0.989	0.472–2.070	0.999	1.001
Main model + Covariate 2								
Collapse-to-FMC interval, min	0.172	0.049	12.434	<0.001	1.188	1.079–1.307	0.269	3.723
Collapse-to-CPR initiation interval, min	−0.047	0.021	5.204	0.023	0.954	0.916–0.993	0.268	3.727
Age, years	0.933	0.486	3.679	0.055	2.541	0.980–6.592	0.936	1.068
Etiology of arrest (non-cardiac vs. cardiac)	2.807	0.509	30.453	<0.001	16.563	6.111–44.889	0.939	1.065
Shockable rhythm	2.024	0.585	11.962	0.001	7.566	2.403–23.817	0.970	1.031

The main model included only the two core time intervals (2 parameters, EPV = 26). Covariate 1 added age (3 parameters, EPV = 17.3). Covariate 2 further added cause of cardiac arrest and presence of a shockable rhythm (5 parameters, EPV = 10.4). Post-exposure interventions (airway management, intravenous access, adrenaline administration, and clinical pathway activation) were not included in the primary analysis, as these variables may act as mediators rather than confounders. Hosmer–Lemeshow test: main model *χ*^2^ = 7.409, df = 8, *P* = 0.493; main model + Covariate 1 *χ*^2^ = 7.415, df = 8, *P* = 0.492; main model + Covariate 2 *χ*^2^ = 8.012, df = 8, *P* = 0.433. EPV, events per variable; ROC, return of spontaneous circulation; CPR, cardiopulmonary resuscitation; VIF, variance inflation factor.

In the main model, the collapse-to-first medical contact interval was significantly associated with increased odds of ROSC (OR 1.136, 95% CI: 1.051–1.228, *P* = 0.001), whereas the collapse-to-CPR initiation interval was not (OR 0.967, 95% CI: 0.931–1.005, *P* = 0.084). After adjustment for age (Model 1), the association remained essentially unchanged (OR 1.136, 95% CI: 1.051–1.229, *P* = 0.001). After further adjustment for age, etiology of arrest, and presence of a shockable rhythm (Model 2), the collapse-to-first medical contact interval remained an independent predictor of ROSC (OR 1.188, 95% CI: 1.079–1.307, *P* < 0.001), and the collapse-to-CPR initiation interval showed a significant inverse association (OR 0.954, 95% CI: 0.916–0.993, *P* = 0.023). Among the covariates in Model 2, non-cardiac etiology (OR 16.563, 95% CI: 6.111–44.889, *P* < 0.001) and presence of a shockable rhythm (OR 7.566, 95% CI: 2.403–23.817, *P* = 0.001) were independently associated with ROSC.

The Hosmer–Lemeshow goodness-of-fit test yielded a *χ*^2^ of 7.409 (df = 8, *P* = 0.493) for the main model, 7.415 (df = 8, *P* = 0.492) for Model 1, and 8.012 (df = 8, *P* = 0.433) for Model 2, indicating satisfactory calibration for all models.

#### Predictive performance of the models

The discriminative ability of the adjusted model and the single time interval was evaluated using receiver operating characteristic (ROC) curve analysis. Bootstrap internal validation with 1,000 resamples yielded an optimism-corrected AUC of 0.882 (95% CI: 0.827–0.936, *P* < 0.001), with a calibration intercept of −0.063, a calibration slope of 0.972, and a Brier score of 0.132, indicating adequate calibration. The optimal probability threshold was 0.4384, corresponding to a sensitivity of 76.9% (95% CI: 65.5–88.4) and a specificity of 87.0% (95% CI: 80.4–93.6), with a Youden index of 0.639 (Figure 2 and Table 6). For the single indicator, the collapse-to-FMC interval yielded an AUC of 0.737 (95% CI: 0.656–0.818, *P* < 0.001), with an optimal cutoff value of 14.992 min, corresponding to a sensitivity of 78.8% (95% CI: 67.7–89.9) and a specificity of 63.0% (95% CI: 53.5–72.5), and a Youden index of 0.418 (Figure 3 and Table 7). These findings suggest that the multivariable model provides good discrimination and calibration (Figure 4), while the single time interval offers moderate predictive value. The collapse-to-FMC interval was selected as the single-indicator predictor in Table 7 because it was the principal exposure of interest in this study, demonstrating a consistent independent association with prehospital ROSC across all multivariable models. The collapse-to-CPR initiation interval was examined in the multivariable models as a secondary time metric to account for the total no-flow period, but the FMC interval was prioritized for single-indicator evaluation given its primary role in the study hypothesis.

**Figure 2:**
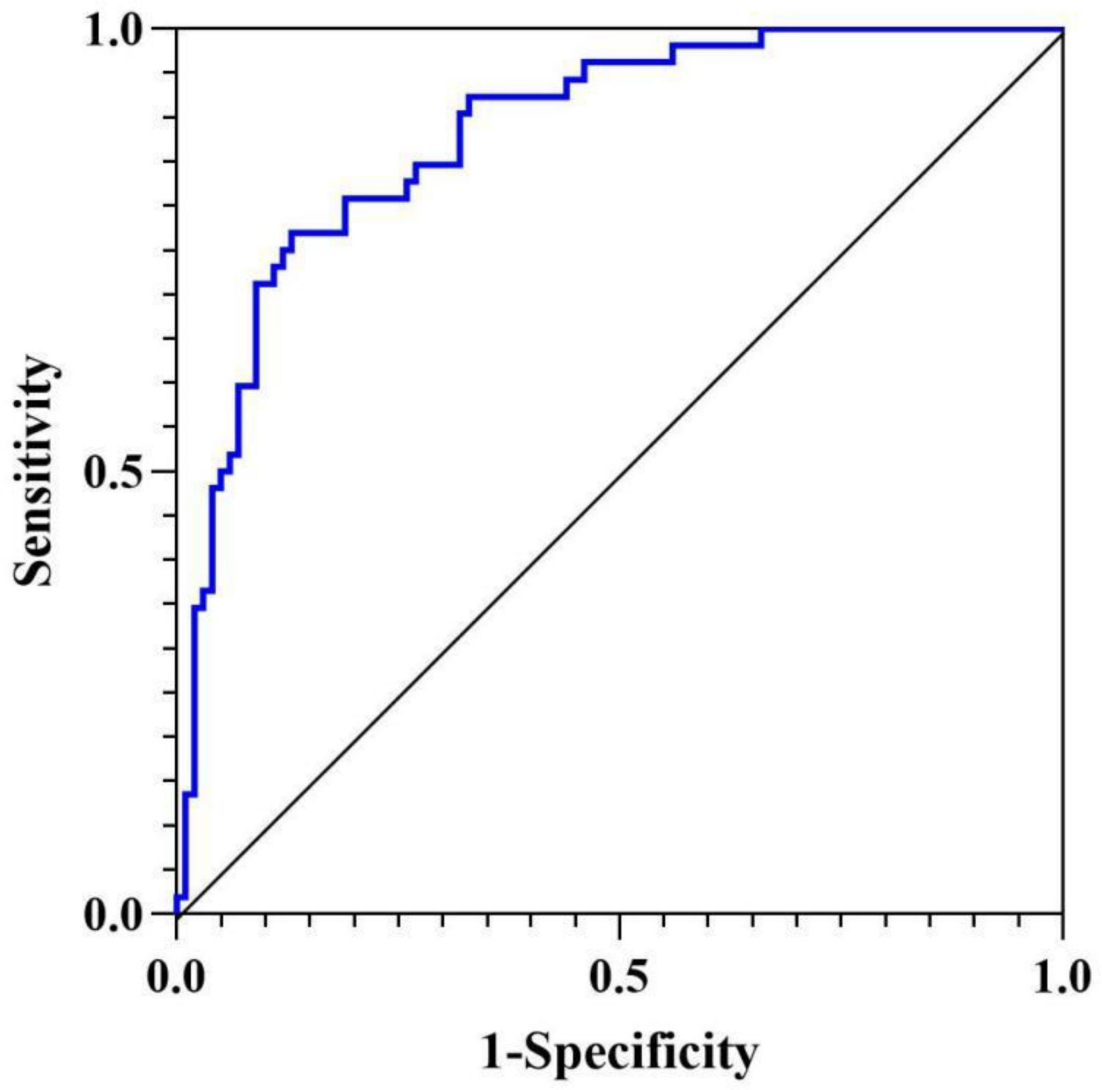
ROC curve of the multivariable model for ROSC prediction. The receiver operating characteristic (ROC) curve demonstrates the discriminative ability of the multivariable model for return of spontaneous circulation (ROSC) in the final analysis cohort (*n* = 152). Bootstrap internal validation with 1,000 resamples yielded an optimism-corrected AUC of 0.882 (95% CI: 0.827–0.936, *P* < 0.001), with a sensitivity of 76.9% (95% CI: 65.5–88.4) and a specificity of 87.0% (95% CI: 80.4–93.6). The optimal probability threshold was 0.4384. OHCA, out-of-hospital cardiac arrest; ROSC, return of spontaneous circulation.

**Figure 3:**
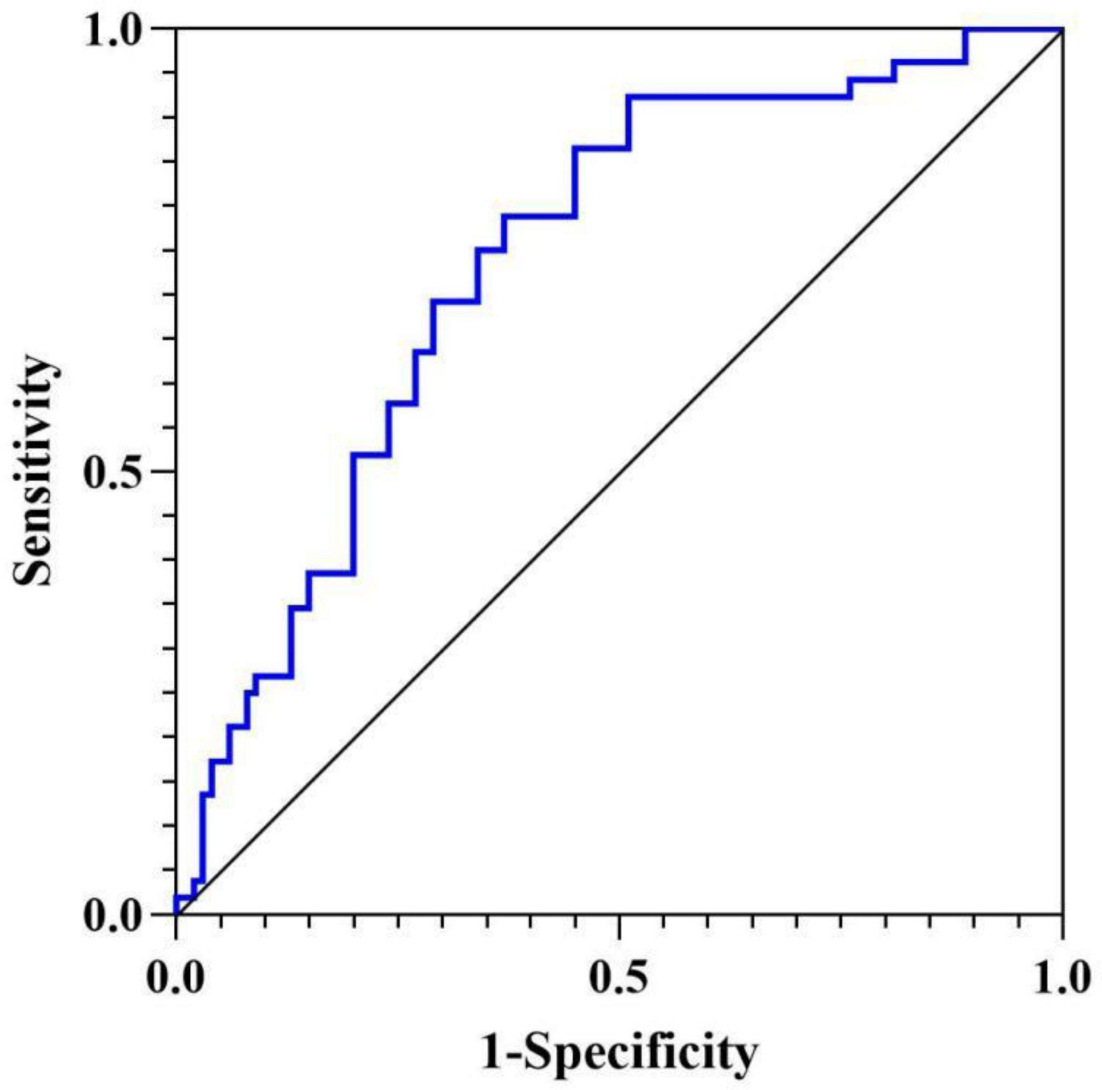
ROC curve of the collapse-to-CPR initiation interval for ROSC prediction. The ROC curve illustrates the predictive performance of the single time interval (from Collapse-to-CPR initiation interval) for ROSC. The optimal cutoff value was 15.767 min, yielding an AUC of 0.737 (95% CI: 0.656–0.817, *P* < 0.001), with a sensitivity of 84.6% and a specificity of 59.0%. OHCA, out-of-hospital cardiac arrest; ROSC, return of spontaneous circulation.

**Figure 4:**
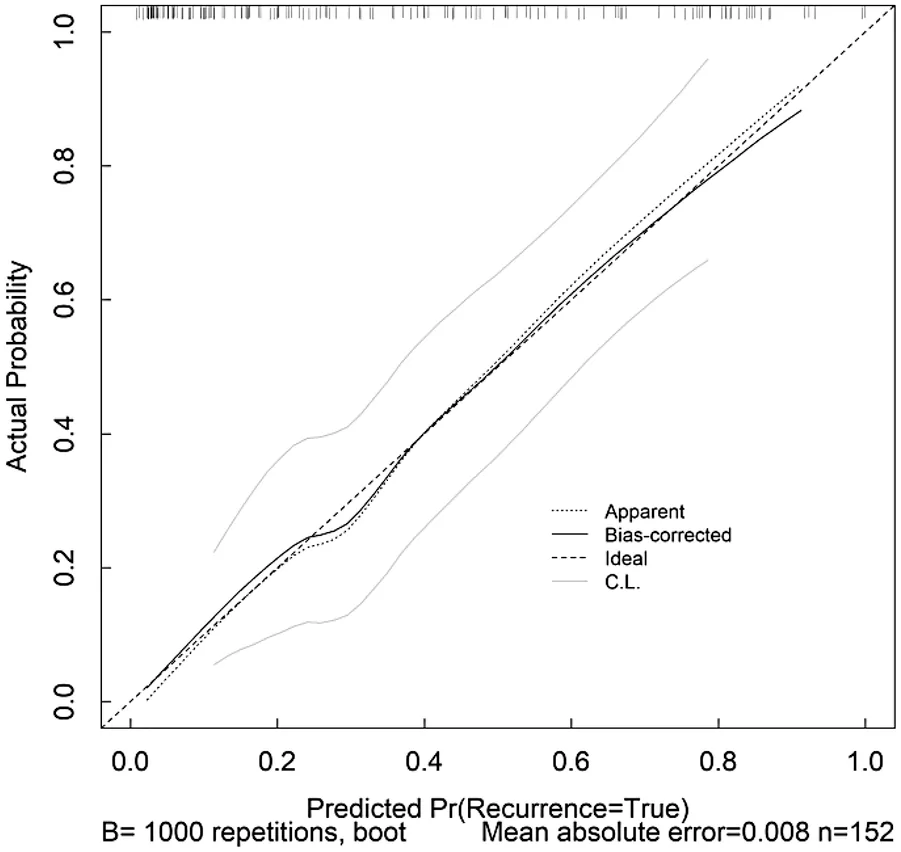
Calibration plot of the adjusted multivariable model for predicting prehospital ROSC. The dashed diagonal line represents perfect calibration. The solid line represents the observed calibration, with a calibration intercept of −0.063 and a calibration slope of 0.972, indicating good agreement between predicted and observed outcomes. Brier score: 0.132. ROSC, return of spontaneous circulation.

**Table 6:** Predictive performance of the multivariable model for ROSC.

Model/Indicator	AUC	95% CI	*P*	Sensitivity	95% CI	Specificity	95% CI	Threshold	Youden index
Adjusted multivariable model	0.882	0.827–0.936	<0.001	76.9%	65.5–88.4	87.0%	80.4–93.6	0.4384	0.639

**Table 7:** Predictive performance of the collapse-to-FMC interval for ROSC.

Model/Indicator	AUC	95% CI	*P*	Sensitivity	95% CI	Specificity	95% CI	Threshold	Youden index
Collapse-to-FMC interval	0.737	0.656–0.818	<0.001	78.8%	67.7–89.9	63.0%	53.5–72.5	14.992	0.418

### Subgroup analyses

Subgroup analyses were conducted to examine whether the association between core time intervals and prehospital ROSC varied across different age groups and etiologies of arrest. Table 8 presents the univariate stratified regression results, and Table 9 presents the multivariable stratified results adjusted for clinical covariates. Formal interaction tests were conducted to assess whether the association between the time intervals and prehospital ROSC differed across subgroups; none reached statistical significance (*P* for interaction >0.05 for all comparisons), indicating that the observed subgroup patterns should be interpreted as exploratory rather than confirmatory.

**Table 8:** Univariate subgroup analyses of core time intervals and prehospital ROSC.

Subgroup	*B*	SE	Wald	*P*	OR	95% CI
Age < 65 years						
Collapse-to-FMC interval, min	0.145	0.053	7.401	0.007	1.156	1.041–1.284
Collapse-to-CPR initiation interval, min	−0.048	0.029	2.704	0.100	0.954	0.901–1.009
Age ≥ 65 years						
Collapse-to-FMC interval, min	0.078	0.069	1.278	0.258	1.081	0.945–1.237
Collapse-to-CPR initiation interval, min	0.019	0.045	0.186	0.666	1.020	0.934–1.113
Cardiac etiology						
Collapse-to-FMC interval, min	0.053	0.070	0.569	0.451	1.054	0.919–1.210
Collapse-to-CPR initiation interval, min	0.068	0.051	1.755	0.185	1.070	0.968–1.184
Non-cardiac etiology						
Collapse-to-FMC interval, min	0.188	0.100	3.549	0.060	1.207	0.992–1.468
Collapse-to-CPR initiation interval, min	−0.107	0.089	1.432	0.231	0.899	0.755–1.070

Each subgroup was analyzed using univariate logistic regression with prehospital ROSC as the dependent variable. OR, odds ratio; CI, confidence interval; CPR, cardiopulmonary resuscitation; ROSC, return of spontaneous circulation.

**Table 9:** Multivariable subgroup analyses of core time intervals and prehospital ROSC.

Subgroup	Variable	*B*	SE	Wald	*P*	OR	95% CI
Age < 65 years	Collapse-to-FMC interval, min	0.119	0.057	4.336	0.037	1.126	1.007–1.260
	Collapse-to-CPR initiation interval, min	−0.013	0.029	0.202	0.653	0.987	0.934–1.044
Age ≥ 65 years	Collapse-to-FMC interval, min	0.534	0.345	2.387	0.122	1.705	0.866–3.355
	Collapse-to-CPR initiation interval, min	−0.049	0.130	0.140	0.708	0.953	0.738–1.229
Cardiac etiology	Collapse-to-FMC interval, min	−0.174	0.164	1.131	0.288	0.840	0.609–1.158
	Collapse-to-CPR initiation interval, min	0.449	0.202	4.934	0.026	1.567	1.054–2.329
Non-cardiac etiology	Collapse-to-FMC interval, min	0.094	0.049	3.673	0.055	1.099	0.998–1.210
	Collapse-to-CPR initiation interval, min	−0.004	0.024	0.029	0.864	0.996	0.950–1.044

Each subgroup was analyzed using the parsimonious modeling approach, restricted to the two core time intervals and prespecified pre-exposure confounders (age, etiology of arrest, and presence of a shockable rhythm) where applicable. OR, odds ratio; CI, confidence interval; CPR, cardiopulmonary resuscitation; ROSC, return of spontaneous circulation.

#### Unadjusted subgroup analyses

In the age-stratified analysis, the collapse-to-first medical contact interval was significantly associated with ROSC among patients younger than 65 years (OR 1.156, 95% CI: 1.041–1.284, *P* = 0.007), whereas no significant association was observed in patients aged 65 years or older (OR 1.081, 95% CI: 0.945–1.237, *P* = 0.258). The collapse-to-CPR initiation interval was not significantly associated with the outcome in either age subgroup (*P* = 0.100 and *P* = 0.666, respectively).

In the etiology-stratified analysis, neither time interval showed a significant association with ROSC among patients with cardiac etiology. Among patients with non-cardiac etiology, the collapse-to-first medical contact interval demonstrated a borderline association with ROSC (OR 1.207, 95% CI: 0.992–1.468, *P* = 0.060), as detailed in Table 8. These findings suggest that the influence of prehospital time intervals on resuscitation outcomes varies by age and etiology of arrest, with a more pronounced effect observed in younger patients and a possible effect in those with non-cardiac causes.

#### Adjusted subgroup analyses

To account for potential confounding, multivariable logistic regression adjusted for clinical covariates was performed within each subgroup. As shown in Table 9, among patients younger than 65 years, the collapse-to-first medical contact interval remained independently associated with ROSC (OR 1.126, 95% CI: 1.007–1.260, *P* = 0.037) after adjustment, whereas the association was not significant in patients aged 65 years or older (*P* = 0.122). Notably, among patients with cardiac etiology, the collapse-to-CPR initiation interval became significantly associated with ROSC after adjustment (OR 1.567, 95% CI: 1.054–2.329, *P* = 0.026), a finding not apparent in the unadjusted analysis. In patients with non-cardiac etiology, the collapse-to-first medical contact interval remained borderline significant (OR 1.099, 95% CI: 0.998–1.210, *P* = 0.055). No significant association was observed for the remaining variables within each subgroup, although wide confidence intervals were noted, likely reflecting the limited sample size in the stratified analyses. Sensitivity analysis comparing complete-case and imputed datasets showed consistent results for age and sex (Table 10). In the complete-case analysis, the median age was 61 years (IQR 50–77) in the non-ROSC group and 62 years (IQR 52.0–75) in the ROSC group (*P* = 0.928), and the proportion of male patients was 71.9% vs. 62.7% (*P* = 0.256). After multiple imputation, the corresponding values were 61 years (IQR 51–74) vs. 61 years (IQR 52.5–73) (*P* = 0.953) and 69.0% vs. 61.5% (*P* = 0.355). The consistent results across complete-case and imputed analyses support the robustness of the imputation approach.

**Table 10:** Sensitivity analysis of age and sex between groups in complete-case and imputed datasets.

Variable	Complete-case analysis				Imputed analysis			
	Non-ROSC	ROSC	*χ*^2^/*z*	*P*	Non-ROSC	ROSC	*χ*^2^/*z*	*P*
Age (years)	61 (50, 77)	62 (52.0, 75)	−0.091	0.928	61 (51, 74)	61 (52.5, 73)	−0.058	0.953
Sex, *n* (%)			1.291	0.256			0.854	0.355
Female	27 (28.13)	19 (37.25)			31 (31.00)	20 (38.46)		
Male	69 (71.88)	32 (62.75)			69 (69.00)	32 (61.54)		

Complete-case analysis included 132 patients for age and 147 patients for sex. Imputed analysis included all 152 patients. Continuous data are presented as median (interquartile range). Categorical data are presented as *n* (%).

Taken together, these results indicate that the association between prehospital time intervals and ROSC is modified by age and etiology of arrest. The interval from collapse to first medical contact appears to be an independent predictor predominantly in younger patients, while the role of the interval from collapse to CPR initiation emerges specifically in patients with cardiac etiology after adjustment for clinical covariates.

## Discussion

This study found that among 152 consecutive patients with out-of-hospital cardiac arrest managed by a single Chinese EMS system, the collapse-to-first medical contact interval was significantly longer in patients who achieved prehospital ROSC than in those who did not (20.0 vs. 13.1 min, *P* < 0.001), and that this association remained independently significant after adjustment for prespecified pre-exposure confounders (adjusted OR 1.188, 95% CI: 1.079–1.307, *P* < 0.001). This appears to contradict the conventional time-to-treatment paradigm in resuscitation medicine. That paradigm derives largely from studies anchored to survival to discharge or favorable neurologic outcome, in which shorter EMS response intervals have been consistently associated with better long-term survival (16, 17). The Utstein guidelines have recommended separate reporting of individual time points because composite time metrics may obscure differences in the intervals they comprise (18, 19). In line with this recommendation, the present study disaggregated the collapse-to-FMC interval from overall response time and adopted prehospital ROSC as the endpoint of interest. The result reveals a relationship that would be masked when intervals are analyzed as a single response-time variable: in this cohort, a longer interval to first medical contact was paradoxically associated with higher odds of ROSC. This finding does not question the value of rapid system response, but it suggests that when the outcome is moved proximally to prehospital ROSC, the direction of the association for a specific interval may be more complex than the traditional assumption of “shorter is always better”.

The longer collapse-to-FMC interval in the ROSC group may be partially explained by differences in case-mix. Patients who achieved ROSC had a substantially higher proportion of non-cardiac etiology (71.2% vs. 22.0%) and a higher prevalence of shockable initial rhythm (30.8% vs. 16.0%). These differences suggest that patients who achieved ROSC may represent a distinct clinical phenotype with greater potential for achieving prehospital ROSC-not greater overall “salvageability” in the sense of hospital survival or intact neurologic recovery. In this manuscript, salvageability is used narrowly: the probability that, given the arrest context, a perfusing rhythm recurs at the prehospital stage. Transient ROSC can precede re-arrest, and ROSC is a proximal physiologic event rather than a patient-centered outcome; whether continued efforts translate into survival or favorable neurologic function depends on arrest etiology, ROSC durability, and post-arrest care (20). This distinction is supported by registry data showing that longer scene time intervals are associated with lower odds of good neurologic recovery even when prehospital ROSC is achieved, reinforcing that prehospital time metrics should not be read as proxies for long-term outcome (21). Moreover, sustained ROSC is a qualitatively distinct endpoint: multivariable models for sustained ROSC emphasize CPR duration, response time, defibrillation, advanced airway, pupil reflex, and glycemic state rather than a single interval from collapse to first contact (22). Our collapse-to-FMC finding therefore speaks to the probability of achieving any prehospital perfusing rhythm, not to the probability of sustained ROSC or hospital survival. Indeed, the duration of resuscitative efforts has been shown to vary according to the perceived potential for salvage, with longer attempts more likely to be pursued in patients judged to have favorable characteristics (23). Previous studies have also shown that the association between prehospital time and outcome is not uniform across etiologies, further supporting the notion that case-mix shapes the apparent effect of time (16, 24, 25). Nevertheless, the persistence of the association after adjustment for age, etiology, and shockable rhythm in the present study suggests that case-mix alone cannot fully account for the observed paradox, and unmeasured confounding related to the severity of the initial arrest or the circumstances of the collapse may have contributed.

The persistence of the independent association after adjustment points to a deeper layer of explanation: the collapse-to-FMC interval may capture unmeasured pathophysiologic heterogeneity. The most informative clue came from the subgroup analysis, in which the collapse-to-CPR initiation interval became significantly associated with ROSC in the cardiac etiology subgroup after adjustment (adjusted OR 1.567, 95% CI: 1.054–2.329, *P* = 0.026). However, the formal interaction test for etiology did not reach statistical significance, and this finding should be regarded as exploratory and hypothesis-generating rather than confirmatory. This is particularly counterintuitive because cardiac arrest of cardiac origin is conventionally considered the most time-critical presentation. A longer interval preceding CPR cannot plausibly confer benefit itself; rather, this pattern may identify a subtype of arrest in which a prolonged agonal phase precedes the loss of effective circulation, witnesses misinterpret the patient's agonal breathing as continued life, and the call for help is consequently delayed. In such patients, the arrest may develop through gradual respiratory or hemodynamic decompensation rather than an abrupt arrhythmic collapse, and the myocardium and brain may tolerate the hypoxic period better than in a sudden arrest of the same nominal duration. Although this interpretation is speculative, it is consistent with the view that distinct pathophysiologic phenotypes of cardiac arrest exist and that the time leading to collapse carries information beyond its duration (26–28). The present findings do not prove this mechanism, but they indicate that the time before initiation of resuscitation can behave differently in different clinical subgroups, a possibility that deserves explicit testing in future studies.

The subgroup findings also carry more direct clinical relevance. Among patients younger than 65 years, the collapse-to-FMC interval remained independently associated with prehospital ROSC after adjustment (adjusted OR 1.126, 95% CI: 1.007–1.260, *P* = 0.037), whereas no significant association was observed in older patients. However, the formal interaction test did not reach statistical significance, and this age-specific pattern should therefore be interpreted as exploratory and hypothesis-generating, requiring confirmation in larger prospective cohorts. This age-dependent pattern is consistent with the greater physiologic reserve and wider tolerable window of ischemia generally attributed to younger cardiac arrest victims (29, 30). The emergence of a significant association for the collapse-to-CPR initiation interval within the cardiac subgroup implies that the pooled analysis may have obscured subgroup-specific signals with opposite directions, and that once the cardiac group is examined separately, time behaves differently than in the non-cardiac group. From a practical perspective, these observations caution against using the reported interval from collapse to CPR initiation as a single decisive criterion for terminating resuscitation. A longer patient-side interval in a young patient or in a patient with cardiac etiology should not automatically be interpreted as denoting futility; rather, it should be weighed together with age, etiology, initial rhythm, and the patient's response to early interventions (31, 32). However, the present study does not provide evidence to construct, support, or modify a termination-of-resuscitation rule. Validated TOR frameworks combine unwitnessed status, lack of bystander CPR, no shock delivery, no prehospital ROSC, and sometimes EMS CPR duration; absence or presence of prehospital ROSC alone is explicitly insufficient (33). The present results thus support only that time intervals should be interpreted within case-mix—not that clinicians may continue or stop resuscitation on the basis of collapse-to-FMC alone.

These findings also inform how prehospital emergency care should be evaluated. In this cohort, conventional dispatch-related intervals did not differ between the ROSC and non-ROSC groups, including EMS arrival time (13.0 vs. 11.9 min, *P* = 0.347) and dispatch-to-scene time (10.0 vs. 9.8 min, *P* = 0.389). Both groups therefore received a comparable speed of system response; what distinguished them was the care delivered after arrival, most notably the rate of clinical pathway activation (98.1% in the ROSC group vs. 70.0% in the non-ROSC group, *P* < 0.001). This suggests that a performance metric fixed solely on dispatch-to-scene response time would fail to capture differences that matter for ROSC. The present findings suggest that prehospital quality improvement should prioritize not only the timeliness of ambulance arrival but also the quality and completeness of the resuscitative care delivered at the scene (34). Concretely, this includes strengthening training in the recognition of potentially reversible etiologies, ensuring the consistent execution of a standardized clinical pathway for patients judged to be salvageable, and building a data infrastructure that links time intervals, interventions, and outcomes in a more granular manner (35). Bystander CPR modality is unlikely to explain our paradox: pooled evidence found that chest-compression-only and standard bystander CPR produce similar prehospital ROSC, survival, and neurologic outcomes in OHCA (36). Thus, the longer collapse-to-FMC association is more plausibly driven by case-mix and resuscitability than by CPR format. This is not to argue that response time is unimportant for OHCA outcome; rather, the message is that speed alone is necessary but not sufficient, and that the quality of on-scene resuscitation deserves at least equal weight in evaluating EMS performance (37).

Several methodologic caveats are emphasized. First, outcome misclassification is possible because prehospital ROSC was recorded as a binary event and did not distinguish transient ROSC (brief perfusing rhythm) from sustained ROSC (≥20 min without chest compressions); some patients with transient ROSC may have re-arrested before hospital arrival, and ROSC itself is less clinically important than survival to discharge (38). Second, resuscitation-duration bias may operate: patients perceived as more salvageable can receive longer on-scene efforts and therefore have greater opportunity to achieve ROSC, which can make longer time exposures appear protective in observational data (38). Third, temporal ambiguity remains for unwitnessed arrests, where collapse time was estimated by the attending physician and the true arrest onset cannot be known exactly. Fourth, residual confounding from unmeasured factors-physician experience, bystander CPR quality, post-ROSC hypotension, lactate, rhythm transitions, and post-arrest carecannot be excluded despite multiple imputation. Although we adopted a DAG-based framework to distinguish pre-exposure confounders from post-exposure mediators, the validity of this framework depends on the accuracy of the assumed causal structure. Fifth, generalizability is limited: this is a single-center EMS cohort with 152 patients and a specific dispatch structure, so findings may not extend to other urban/rural systems, BLS/ALS configurations, or jurisdictions with different TOR policies (39).

Several limitations of this study should be acknowledged. First, this was a single-center retrospective analysis with a modest sample size of 152 patients. To mitigate sparse-data bias, we adopted a clinically prespecified parsimonious modeling strategy with a maximum of five parameters (EPV = 10.4), and all subgroup analyses were restricted to the core time intervals and prespecified confounders. The wide confidence intervals in some subgroup analyses indicate limited statistical precision due to the modest number of events within individual strata, and these results warrant cautious interpretation. Regarding the primary multivariable models, the events-per-variable ratios ranged from 26 (main model) to 10.4 (Model 2), all exceeding the recommended threshold of 10. Bootstrap internal validation with 1,000 resamples demonstrated minimal optimism (optimism-corrected AUC 0.882, 95% CI: 0.827–0.936) and a calibration slope close to 1 (0.972), suggesting that the estimated odds ratios and confidence intervals in the primary models were stable and not substantially inflated by overfitting. Nevertheless, given the modest sample size and the observational design, the precise estimates should be interpreted with appropriate caution, and external validation in larger cohorts is warranted. Second, the time of collapse was determined from interviews with family members or bystanders and is therefore susceptible to recall bias (40); if such bias occurred, it may have systematically prolonged the estimated collapse-to-FMC interval in the ROSC group. Third, several potentially important confounders, such as the clinical experience of individual physicians, family attitudes toward prolonged resuscitation, and the patient's baseline functional status could not be measured or adjusted for (41). Multiple imputation was used to handle missing covariate data, but this cannot eliminate the effect of unobserved factors (42). Fourth, the primary outcome was prehospital ROSC, and no information was available on survival to hospital discharge or neurologic status. Because prehospital ROSC is an intermediate endpoint, the direction of the observed association may not be directly generalizable to long-term outcomes, and future studies with complete long-term follow-up are needed. Finally, given the observational design, no causal relationship can be inferred. The most reasonable reading of the data is that a longer collapse-to-FMC interval functions as a composite marker that reflects a particular case-mix and possibly a distinct pathophysiologic subtype of arrest, none of which implies that delaying contact with the patient improves outcome in itself (43). Within these limitations, the present study provides evidence from a Chinese single-system EMS cohort that the collapse-to-FMC interval is independently associated with prehospital ROSC in a direction opposite to the traditional time-critical assumption. This paradox suggests that prehospital time metrics should not be reduced to a single universal target, but should be interpreted in the context of arrest etiology, patient age, and the quality of the resuscitative effort. Future multicenter investigations that capture detailed time-event sequences, physiologic data, and long-term survival are required to clarify the mechanisms underlying these findings and to guide the design of resuscitation protocols that are both timely and targeted.

## Conclusion

This study evaluated the association between prehospital time intervals and resuscitation outcomes in patients with out-of-hospital cardiac arrest managed by a regional EMS system. Conventional dispatch-to-arrival metrics were not associated with prehospital return of spontaneous circulation, whereas the collapse-to-first medical contact interval remained independently associated with the outcome after adjustment for prespecified pre-exposure confounders, particularly in younger patients. The direction of this association, with longer intervals observed in survivors, was contrary to expectations, suggesting that this time interval may reflect a prehospital-ROSC phenotype and unmeasured case-mix characteristics rather than treatment delay *per se*. These findings apply to the intermediate endpoint of prehospital ROSC and must not be extended to survival, neurologic outcome, or termination-of-resuscitation policy without prospective validation. From a clinical standpoint, our findings highlight the importance of prompt identification of reversible etiologies and standardized resuscitation care, rather than merely shortening response times. Future prospective multi-center studies are needed to clarify the causal relationship between time intervals and prehospital outcomes.

## Data Availability

The raw data supporting the conclusions of this article will be made available by the authors, without undue reservation.
